# Modulation of BCR Signaling by the Induced Dimerization of Receptor-Associated SYK

**DOI:** 10.3390/antib6040023

**Published:** 2017-12-07

**Authors:** Mark L. Westbroek, Robert L. Geahlen

**Affiliations:** Department of Medicinal Chemistry and Molecular Pharmacology, Purdue Center for Cancer Research, Purdue University, West Lafayette, IN 47907, USA; westbroe@purdue.edu

**Keywords:** BCR signaling, SYK, chemical inducer of dimerization, NFAT, calcium mobilization

## Abstract

Clustering of the B cell antigen receptor (BCR) by polyvalent antigens is transmitted through the SYK tyrosine kinase to the activation of multiple intracellular pathways that determine the physiological consequences of receptor engagement. To explore factors that modulate the quantity and quality of signals sent by the crosslinked BCR, we developed a novel chemical mediator of dimerization to induce clustering of receptor-associated SYK. To accomplish this, we fused SYK with *E. coli* dihydrofolate reductase (eDHFR), which binds the small molecule trimethoprim (TMP) with high affinity and selectivity and synthesized a dimer of TMP with a flexible linker. The TMP dimer is able to induce the aggregation of eDHFR-linked SYK in live cells. The induced dimerization of SYK bound to the BCR differentially regulates the activation of downstream transcription factors, promoting the activation of Nuclear Factor of Activated T cells (NFAT) without affecting the activation of NFκB. The dimerization of SYK enhances the duration but not the amplitude of calcium mobilization by enhancing the extent and duration of its interaction with the crosslinked BCR at the plasma membrane.

## 1. Introduction

The B cell antigen receptor (BCR) is a macromolecular complex consisting of a membrane-bound immunoglobulin and the heterodimeric co-receptors CD79A and CD79B [1]. The cytoplasmic tails of CD79A and CD79B each contain two conserved tyrosines that constitute part of an immunoreceptor tyrosine-based activation motif (ITAM) [2]. Binding of antigen to the BCR results in rapid ITAM phosphorylation, which then serves as a scaffold for docking of signaling components [2,3,4]. Although the mechanism by which binding of antigen initiates phosphorylation of ITAM tyrosines is not well understood, several models seek to explain the phenomenon. The most widely accepted model is the BCR clustering model, where binding of polyvalent antigens induces clustering of BCR complexes [5,6], thus bringing signaling components into close enough proximity to activate their functions. This was first proposed due to experiments that demonstrated that divalent anti-BCR antibodies were able to activate BCR signaling, while monovalent Fab fragments were not [7,8,9,10,11]. A second suggested model is that antigen binding induces a conformational change in the BCR that ultimately initiates signaling [12]. More recent research points to a third model called the dissociation-activation model. This proposes that the BCR exists as auto-inhibited oligomers, or microclusters, that are opened and activated upon the binding of antigen [13,14]. Thus, actin depolymerization alone, which mimics the transient actin depolymerization that is induced by BCR engagement, induces signaling by opening the BCR oligomer even without BCR crosslinking [15,16,17,18].

Despite the differences in these models, they all agree that the extent of B cell activation relies on the receptor affinity for the antigen, as well as antigen dose and valency [6]. Recent studies using synthetic antigens have demonstrated that while low valency antigens are capable of activating BCR signaling, high valency antigens promote a greater degree of signaling and successfully induce production of antibodies in vivo [6,19,20]. Thus, more BCR crosslinking induces a greater response. 

Both signaling and aggregation of antigen-bound receptors are enhanced by intracellular components. Specifically, the non-receptor tyrosine kinase SYK promotes a positive feedback loop to enhance ITAM phosphorylation and signaling [21,22]. SYK consists of two N-terminal SH2 domains and a C-terminal kinase domain [1]. Binding of the tandem SH2 domains to doubly phosphorylated ITAMs confers a conformational change that activates SYK and allows it to phosphorylate downstream signaling proteins [23]. Studies have shown that, upon binding to the doubly phosphorylated ITAM, SYK is able to phosphorylate neighboring ITAMs to recruit more SYK and enhance signaling in a positive feedback loop [22]. Additionally, SYK binding to the phosphorylated ITAM further activates the receptor through an inside-out signaling mechanism to amplify signaling [21].

At the termination of signaling, the BCR complex is internalized via a clathrin- and actin-dependent mechanism and the antigen is processed for presentation by MHC class II complexes [24,25]. The receptor proximal tyrosine kinase LYN and SYK differentially modulate receptor internalization. While LYN is required for internalization [26], SYK prolongs BCR residence at the plasma membrane. The BCR of SYK-deficient cells is rapidly internalized upon antigen engagement, while the BCR of SYK-sufficient cells remains clustered at the membrane [26]. SYK inhibition results in rapid BCR internalization [27]. 

We hypothesized, therefore, that enhancing the interaction between SYK molecules associated with clustered BCR complexes would augment receptor retention at the membrane. To investigate this, we developed and employed a novel chemical inducer of dimerization to selectively aggregate SYK when bound to the BCR. We demonstrate that SYK dimerization does modulate BCR signaling and increase the retention of kinase-BCR complexes at the plasma membrane. Interestingly, this induced dimerization plays a selective role in enhancing signaling from the receptor to the activation of NFAT.

## 2. Materials and Methods

### 2.1. Plasmids, DNA Constructs and Antibodies

The Myc-SYK construct was generated as previously described [28]. Myc-SYK and Myc-epitope tag cDNAs were subcloned into the pLL-1 vector (Active Motif, Carlsbad, CA, USA) to generate Myc-SYK-eDHFR and Myc-eDHFR constructs. These were then further subcloned into the pCDH-CMV-MCS-EF1-Puro lentiviral vector (System Biosciences, Palo Alto, CA, USA). NLyn-SYK-eDHFR was generated by cloning SYK with a forward primer coding for the first five N-terminal residues of LYN and inserting the construct into the pLL-1 vector.

The following antibodies were used for Western blotting and/or immunofluorescence: SYK (D3Z1E XP, Cell Signaling Technology, Danvers, MA, USA), Myc (9B11, Cell Signaling Technology, Danvers, MA, USA), G3BP (611126, BD Biosciences, San Jose, CA, USA) and GAPDH (AM4300, Ambion, Austin, TX, USA). HRP-coupled secondary antibodies were purchased from Pierce and AlexaFluor 405- and 594-coupled secondary antibodies from Invitrogen (Carlsbad, CA, USA).

### 2.2. Cell Culture and DNA Transfection/Transduction

HEK293T (ATCC) and SYK-deficient MCF7 (MCF7-BD) cells [29] were cultured in DMEM medium supplemented with 10% FBS, 0.37% NaHCO_3_, 100 IU/mL penicillin and 100 µg/mL streptomycin. SYK-deficient DT40 cells [30] were cultured in RPMI 1640 medium supplemented with 10% FBS, 1% chicken serum, 50 µM 2-mercaptoethanol, 1 mM sodium pyruvate, 100 IU/mL penicillin G and 100 µg/mL streptomycin.

DT40 cells were transfected via electroporation. The cells were collected (1 × 10^7^) and resuspended in 500 µL cell culture medium and incubated with the respective plasmids at room temperature for 5 min. Cells were electroporated at 250 V, 975 µF and were incubated on ice for 10 min. Cells were then recovered in 10 mL pre-warmed cell culture medium for 24–48 h.

To generate cells stably expressing Myc-SYK-eDHFR or Myc-eDHFR, lentiviral particles were packaged using HEK293T cells. HEK293T, SYK-deficient DT40 and MCF7-BD cells were transduced with the lentiviral particles, selected with puromycin (1 µg/mL) and screened for expression by Western blotting.

### 2.3. TMP-Agarose Pull-Down

HEK293T cells stably expressing Myc-SYK-eDHFR or Myc-eDHFR were grown to about 70% confluence on 10 cm culture dishes. Cells were lysed with ice-cold 50 mM Tris, pH 7.5, 150 mM NaCl, 10% glycerol, 1% NP-40, 1 mM sodium orthovanadate, 1× protease inhibitor cocktail (Sigma-Aldrich, St. Louis, MO, USA, p8340), sonicated and centrifuged at 17,000× *g* for 5 min at 4 °C. Lysates were pre-cleared by incubating with Sepharose beads (30 µL) at 4 °C for 30 min. Lysates were then incubated with TMP-agarose beads (5 µL) plus Sepharose beads (30 µL) for 15 min at 4 °C. The beads were washed three times with lysis buffer and transferred to new tubes with the final wash. SDS-sample buffer (200 µL) was added. Samples were heated for 5–10 min at 99 °C, separated by SDS-polyacrylamide gel electrophoresis and immunoblotted for the Myc-epitope.

### 2.4. Immunofluorescence

MCF7-BD cells stably expressing Myc-SYK-eDHFR or Myc-eDHFR were plated on coverslips in 6-well plates and treated with MG132 (100 µM, Selleck Chemicals, Houston, TX, USA) with or without TMP dimer for 6 h at 37 °C and then immunostained for the stress granule marker G3BP. To demonstrate SYK localization, SYK-deficient DT40 cells were transiently transfected with NLyn-SYK-eDHFR or Myc-SYK-eDHFR plasmids and plated on poly-l-lysine-coated coverslips. They were then immunostained for SYK. For BCR retention assays, DT40 cells stably expressing Myc-SYK-eDHFR were plated on poly-l-lysine-coated coverslips and treated with Texas Red-labeled goat anti-chicken IgM (Rockland, Gilbertsville, PA, USA) for the indicated times. The cells were then immunostained for SYK.

### 2.5. NFAT- and NFκB-Luciferase Reporter Assays

SYK-deficient DT40 cells were electroporated with 30 µg of the indicated eDHFR-linked protein expression plasmid or mock plasmid and 10 µg NFAT- or NFκB-luciferase plasmid and then allowed to recover for 24–48 h. The cells were collected and resuspended in medium lacking chicken serum at a density of 1 × 10^6^ cells/mL. Cells were treated with either 1 µM ionomycin plus 50 ng/mL PMA, 0.5% dimethylsulfoxide (DMSO), 0.5 µg/mL goat anti-chicken IgM (Bethyl Laboratories, Montgomery, TX, USA), anti-IgM plus 2 µM Latrunculin B (LatB, AdipoGen, San Diego, CA, USA), or anti-IgM plus the indicated concentration of TMP dimer for 6 h at 37 °C. Luciferase activity was measured using a Promega Luciferase Assay System kit per manufacturer instructions. All measured values were normalized to the PMA/ionomycin internal control. All experiments consisted of three technical replicates and were repeated 3–4 times.

### 2.6. Intracellular Calcium Assay

Intracellular calcium was measured using a Fluo-4 NW Calcium Assay Kit (Invitrogen, Carlsbad, CA, USA) per manufacturer’s protocol. Briefly, DT40 cells stably expressing Myc-SYK-eDHFR were collected and resuspended in assay buffer at 2 × 10^7^ cells/mL. Cells were aliquoted to a 96-well plate (50 µL/well) and incubated at 37 °C for 1 h. Fluo-4 2× dye was then added (50 µL/well) and the cells were incubated at 37 °C for 30 min and then 25 °C for 30 min. Background fluorescence was measured. Then 25 µL of 5× anti-IgM (10 µg/mL final concentration) or anti-IgM plus TMP dimer (10 µM final concentration) was auto-injected. Fluorescence readings were measured every 5 s over 5 min on a BioTek Synergy4 plate reader. Each treatment condition was repeated in triplicate and on three different days. Background fluorescence was subtracted, replicates were averaged and all values were normalized to peak values.

### 2.7. ImageJ Analyses

Stress granule size was measured using the ImageJ Analyze Particles function (> Analyze > Analyze Particles) after subtracting background (> Process > Math > Subtract) and adjusting automated color threshold (> Image > Adjust > Color Threshold). Cutoff size was set to 5-Infinity. The stress granules ≥150 pixel^2^ in each image were counted and divided by the number of 4’,6-diamidino-2-phenylindole (DAPI)-stained nuclei in the same field to calculate stress granules/cell. Colocalization was analyzed using the ImageJ Coloc2 function (> Analyze > Colocalization > Coloc2). The Pearson correlation coefficient was used to measure colocalization by quantitatively scoring the correlation between the two fluorophores.

### 2.8. Synthesis of TMP Coupled to Agarose Beads

The 4′-methoxy group of TMP was cleaved as previously described [31]. TMP (10.0 g, 34.5 mmol) was dissolved in 48% hydrobromic acid (120 mL) which had been preheated to 95 °C and stirred at 95–100 °C for 20 min. The heat bath was then removed and sodium hydroxide (50% aq., 30 mL) was added dropwise. Stirring was then stopped and the reaction mixture was allowed to cool to room temperature over 2 h, yielding a white precipitate. Precipitated crystals were filtered, washed with ice-cold water and allowed to mostly dry in Buchner funnel. They were then dissolved in boiling water (150 mL) and neutralized to pH 7 by slow addition of aq. ammonium hydroxide (30% *w*/*w*). This solution was then placed at 4 °C overnight, resulting in crystal precipitation. The precipitate was then filtered, rinsed with ice-cold water and dried in vacuo, yielding **1** as beige crystals (4.99 g, 53%). ^1^H NMR (300 MHz, CD_3_OD) δ ppm: 7.21 (s, 1H), 6.54 (s, 2H), 3.82 (s, 6H), 3.63 (s, 2H).

5-Iodovalerate (**2**) was prepared as previously described [32], with slight modification. Sodium iodide (6.16 g, 41.1 mmol) was dissolved in dry acetone (26 mL) and brought to reflux. Ethyl 5-bromovalerate (5.0 mL, 31.6 mmol) was then added and the reaction was stirred at reflux for 3 h. Additional sodium iodide (2.37 g, 15.8 mmol) was then added and the solution was stirred at reflux for 1 h. The reaction was cooled and 13 mL ether was added. The solution was filtered and solvent evaporated in vacuo. The residue was dissolved in 40 mL ether and then washed twice each with 12–13 mL of 1% NaOH (aq), ddH_2_O and brine. The organic fraction was then dried over sodium sulfate and the solvent removed in vacuo, yielding a pale-yellow oil (3.76 g, 93%). ^1^H NMR (300 MHz, CDCl_3_) δ ppm: 4.01 (q, 2H), 3.08 (t, 2H), 2.22 (t, 2H, 1.75 (m, 2H), 1.64 (m, 2H), 1.15 (t, 3H).

To generate the 4′-substituted phenol **4**, **1** (2.00 g, 7.24 mmol), **2** (2.04 g, 7.97 mmol) and cesium carbonate (4.72 g, 14.48 mmol) were dissolved in dimethylformamide (DMF) (180 mL) and stirred at 70 °C for 6 h. Solvent was then removed in vacuo. The crude product was directly purified by flash silica gel chromatography (DCM→10% MeOH/DCM), yielding a brown solid (1.55 g, 53%). **3** (1.55 g, 3.82 mmol) was then dissolved in MeOH (7.6 mL) and NaOH (5 M, 2.30 mL, 11.5 mmol) was added. The reaction was stirred at room temperature for 1 h. The solvent was then removed, water (26.0 mL) was added and acidified to pH 4 with 1 M HCl. The precipitate was filtered, rinsed with ice-cold water and dried in vacuo, yielding a light brown solid (0.94 g, 66%). ^1^H NMR (300 MHz, DMSO-*d*_6_) δ ppm: 7.48 (s, 1H), 6.53 (s, 2H), 6.34 (s, 2H), 5.95 (s, 2H), 3.76 (t, 2H), 3.70 (s, 6H), 3.52 (s, 2H), 2.24 (t, 2H), 1.61 (m, 4H).

To prepare the immobilized TMP ligand **5**, **4** (8.0 mg, 21 µmol) and 1,1′-carbonyldiimidazole (4.4 mg, 27 µmol) were dissolved in DMF (1 mL) and mixed at 50 °C, 1000 rpm for 30 min in an Eppendorf Thermomixer (Eppendorf, Hauppauge, NY, USA). CarboxyLink^TM^ Coupling Gel (Thermo Fisher, Rockford, IL, USA) (1 mL) was washed with DMF (1 mL × 3), then combined with the activated acid and mixed overnight at 50 °C, 1000 rpm. The beads were then centrifuged and solvent removed. Coupling efficiency was monitored by UV absorbance of solvent before and after reaction and determined to be 50–60%. The beads were washed with DMF (1 mL × 3) and *N*-acetoxysuccinimide (1 mL of 1 M solution in DMF) was added to the beads and mixed for 2 h at room temperature to protect residual free amines. The beads were then washed with DMF (1 mL × 3) and PBS (1 mL × 3) and resuspended in 0.05% NaN_3_ in PBS (500 µL) to make a 50% slurry. To prepare *N*-acetoxysuccinimide, *N*-hydroxysuccinimide (0.50 g, 4.35 mmol) and acetic anhydride (1.24 mL, 13.1 mmol) were combined and stirred overnight at room temperature. The solvent was removed. Crystals were filtered and rinsed with hexanes and dried in vacuo, yielding white crystals (0.637 g, 93%). ^1^H NMR (CDCl_3_, 300 MHz) δ ppm: 2.82 (s, 4H), 2.32 (s, 3H).

### 2.9. Synthesis of TMP Dimer 6

**1** (0.500 g, 1.33 mmol) and 1,1′-carbonyldiimidazole (0.260 g, 1.60 mmol) were dissolved in DMF (26 mL) and stirred at room temperature for 30 min. Diethylenetriamine (72 µL, 0.66 mmol) was then added and the reaction was stirred at room temperature for ~24 h. The solvent was then removed in vacuo. The crude product was purified by HPLC (5%→25% ACN/H_2_O over 1 h) and fractions appearing to contain product were screened by MALDI MS. Fractions containing pure product were pooled and lyophilized. ^1^H NMR (300 MHz, DMSO-*d*_6_) δ ppm: 7.22 (s, 2H), 6.56 (s, 4H), 3.91 (s, 4H), 3.80 (s, 12H), 3.66 (s, 4H), 3.49 (m, 4H), 3.18 (m, 4H), 2.36 (m, 4H), 1.85 (m, 4H), 1.72 (m, 4H). MALDI MS (m + H): 820.4413 (calculated: 819.95)

## 3. Results

### 3.1. eDHFR-Tagged SYK Binds TMP

**Figure 1:**
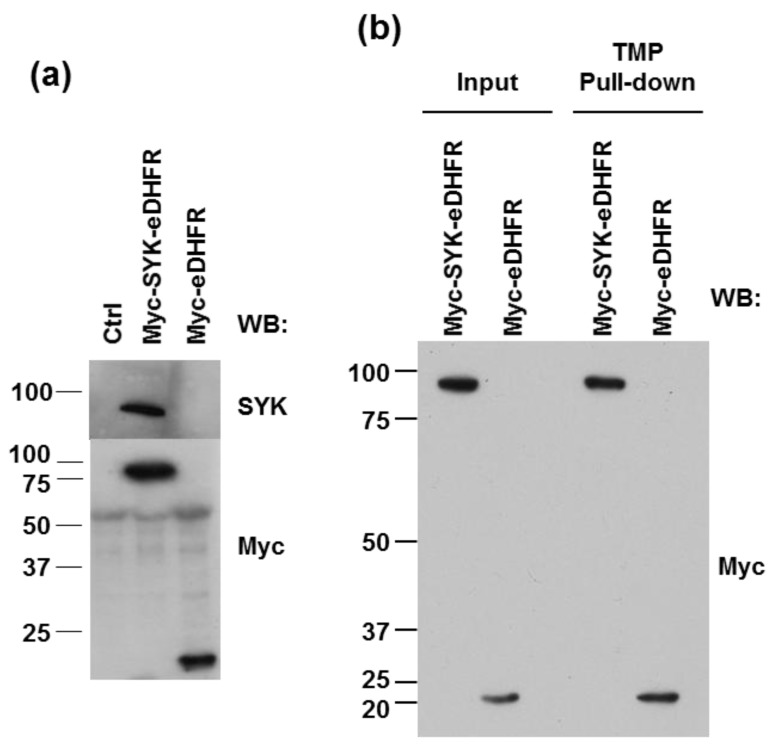
eDHFR-linked fusion proteins are stable and bind trimethoprim (TMP). (**a**) HEK293T cells were transfected with either recombinant Myc-SYK-eDHFR or control Myc-eDHFR plasmids and stable transfectant cell lines were produced. Western blot (WB) analysis of lysates from control HEK293T cells (Crtl) or stable transfectant HEK293T cells using antibodies against SYK or the Myc-epitope tag (Myc); (**b**) Lysates from HEK293T cells expressing Myc-SYK-eDHFR or Myc-eDHFR were adsorbed to immobilized TMP, shown in Figure 2a. Proteins in lysates (Input) or bound to beads were detected by Western blotting using anti-Myc epitope (Myc) antibodies.

We sought to develop a process for inducing the dimerization of SYK in intact cells by first generating a fusion protein containing a subunit with small molecule binding capabilities. For this, we created expression constructs coding for proteins fused with *E. coli* dihydrofolate reductase (eDHFR). eDHFR is a small protein of 18 kDa that binds the small molecule trimethoprim (TMP), a competitive inhibitor, with very high affinity (K_d_ = 9.1 nM). TMP can be selectively modified without significantly affecting its binding [33,34]. We generated constructs for the expression of a Myc-epitope tagged form of SYK (Myc-SYK-eDHFR) and, as a control, Myc-eDHFR. Transfections in HEK293T cells indicated that both forms could be stably expressed (Figure 1a). 

**Figure 2:**
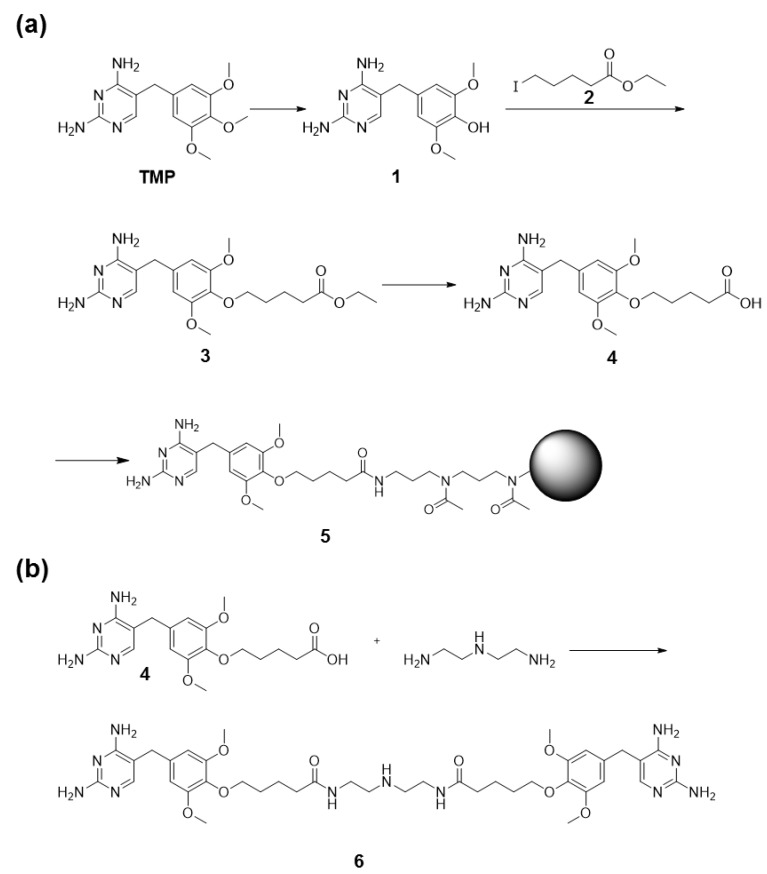
Preparation of derivatives of trimethoprim (TMP). (**a**) Reaction scheme for the preparation of TMP covalently coupled to agarose beads; (**b**) Reaction scheme for the preparation of a TMP dimer.

To determine if eDHFR-tagged proteins retained the ability to bind TMP, we synthesized a form of TMP covalently bound to agarose beads using the scheme outlined in Figure 2a. These were used in pull-down experiments to isolate tagged proteins from lysates of HEK293T cells stably expressing either Myc-SYK-eDHFR or Myc-eDHFR. As shown in Figure 1b, both proteins were effectively pulled down by these beads, demonstrating the ability of the tagged proteins to bind TMP.

### 3.2. TMP Dimer Induces SYK Aggregation

**Figure 3:**
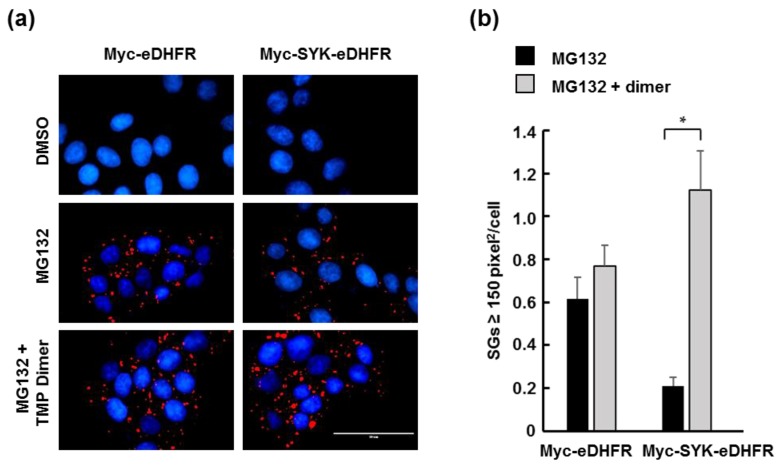
The size of cellular ribonuclear protein aggregates increased when SYK and trimethoprim (TMP) dimerizing agent were present. (**a**) MCF7-BD cells stably expressing Myc-eDHFR or Myc-SYK-eDHFR were treated with or without MG132 to induce the formation of SGs in the presence or absence of TMP dimer. Cells were fixed and stained with antibodies against G3BP (red) and stained with 4’,6-diamidino-2-phenylindole (DAPI) to visualize nuclei (blue). Bar, 50 μm; (**b**) Large stress granules (SGs) were counted in cells treated as described in panel a. The histogram represents average ± standard error of the mean (SEM) of 80–100 cells from experiments repeated three times. * *p* < 0.05. DMSO, dimethylsulfoxide.

To generate a small molecule dimerizer of eDHFR-tagged proteins, we coupled two molecules of TMP together, separated by a 17-atom spacer (Figure 2b). To validate that this TMP dimer was capable of inducing SYK aggregation, we took advantage of a known interaction to visualize SYK when present in a large complex in live cells. SYK is recruited to ribonucleoprotein particles called stress granules (SGs), which accumulate in cells treated with the proteasome inhibitor MG132 [35,36]. SG formation occurs independently of the presence or activity of SYK in MCF7 cells [36]. Utilizing this system, we induced SG formation in MCF7-BD cells stably expressing Myc-SYK-eDHFR or Myc-eDHFR in the absence or presence of added TMP dimer to determine if dimerization could promote the formation of larger droplets. With addition of the TMP dimer, the number of large SGs (≥150 pixel^2^) per cell showed a five-fold increase in cells expressing Myc-SYK-eDHFR but remained unchanged in cells expressing Myc-eDHFR (Figure 3). 

### 3.3. Dimerized SYK Signals through NFAT or NFκB When Interacting with the BCR

**Figure 4:**
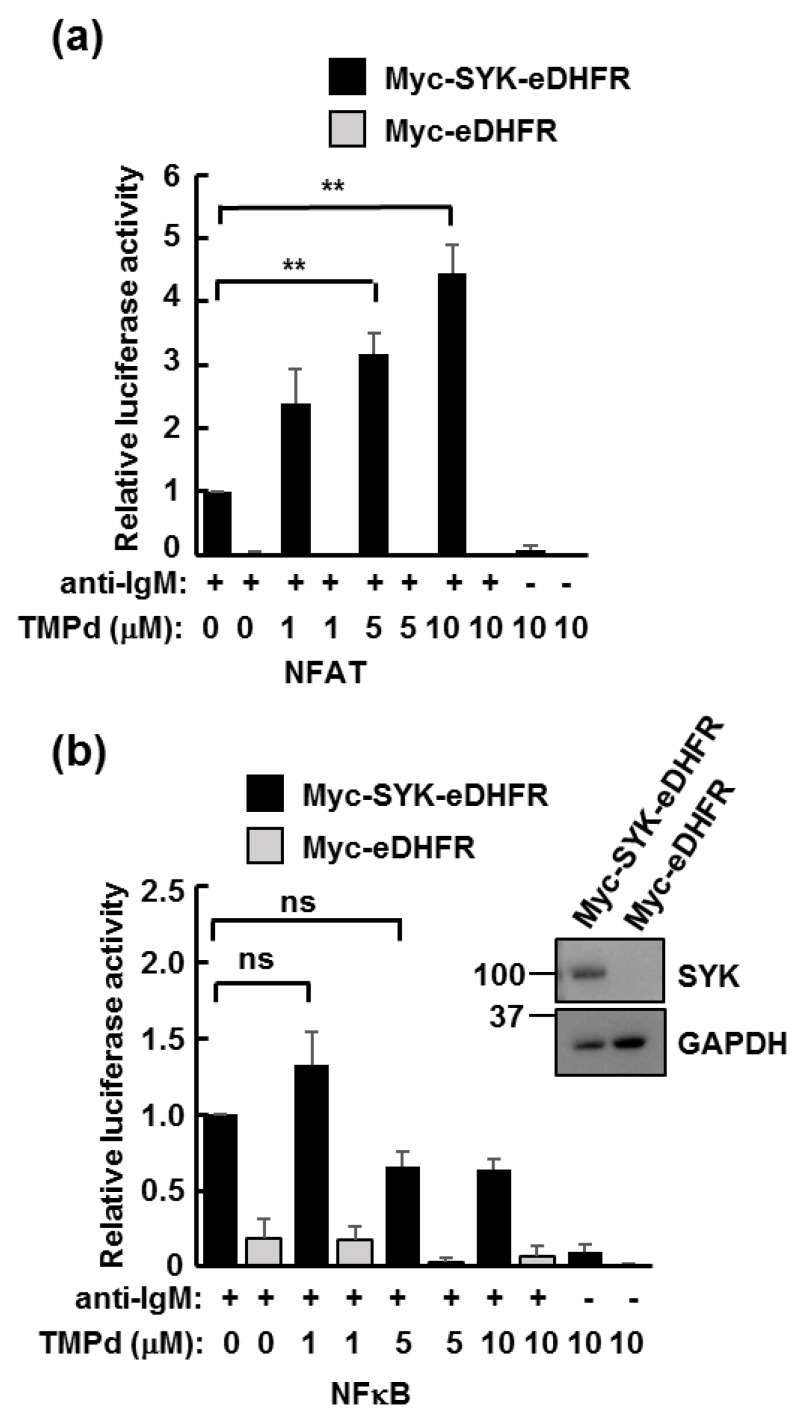
The trimethoprim (TMP) dimer enhances B cell antigen receptor (BCR)-dependent activation of Nuclear Factor of Activated T cells (NFAT) but not NFκB. (**a**) SYK-deficient DT40 cells transiently transfected with an NFAT-driven luciferase reporter plasmid and with plasmids for the expression of either Myc-eDHFR or Myc-SYK-eDHFR were activated by receptor crosslinking with anti-IgM where indicated in the presence of increasing concentrations of TMP dimer (TMPd). Values were normalized to 1 for cells activated with anti-IgM in the absence of TMPd. Values represent means ± standard error of the mean (SEM) of triplicate assays repeated three times. ** *p* < 0.01; (**b**) SYK-deficient DT40 cells were transfected and treated as in panel a, except that an NFκB-driven luciferase reporter plasmid was used. Values represent means ± SEM of triplicate assays repeated three times ns, not significant. The expression of Myc-SYK-eDHFR was verified by Western blotting with antibodies against SYK. Glyceraldehyde-3-phosphate dehydrogenase (GAPDH) was detected as a loading control.

To determine if Myc-SYK-eDHFR could support BCR signaling, we expressed the protein in SYK-deficient B cells and employed luciferase reporter assays for two transcription factors that are activated downstream of BCR signaling: NFAT and NFκB. Myc-SYK-eDHFR was able to propagate signals from the BCR to both transcription factors. Myc-eDHFR, which was used as a control, was inactive as expected (Figure 4). 

We explored the effects of induced SYK dimerization on BCR signaling by altering the extent to which the SYK containing complexes were clustered by treating cells with anti-IgM to crosslink the BCR and titrating in different amounts of TMP dimer. Interestingly, titration of higher concentrations of TMP dimer resulted in significantly increased NFAT-luciferase activity induced by anti-IgM (Figure 4a). However, addition of the dimerizer had no significant effect on BCR-stimulated NFκB activity (Figure 4b).

**Figure 5:**
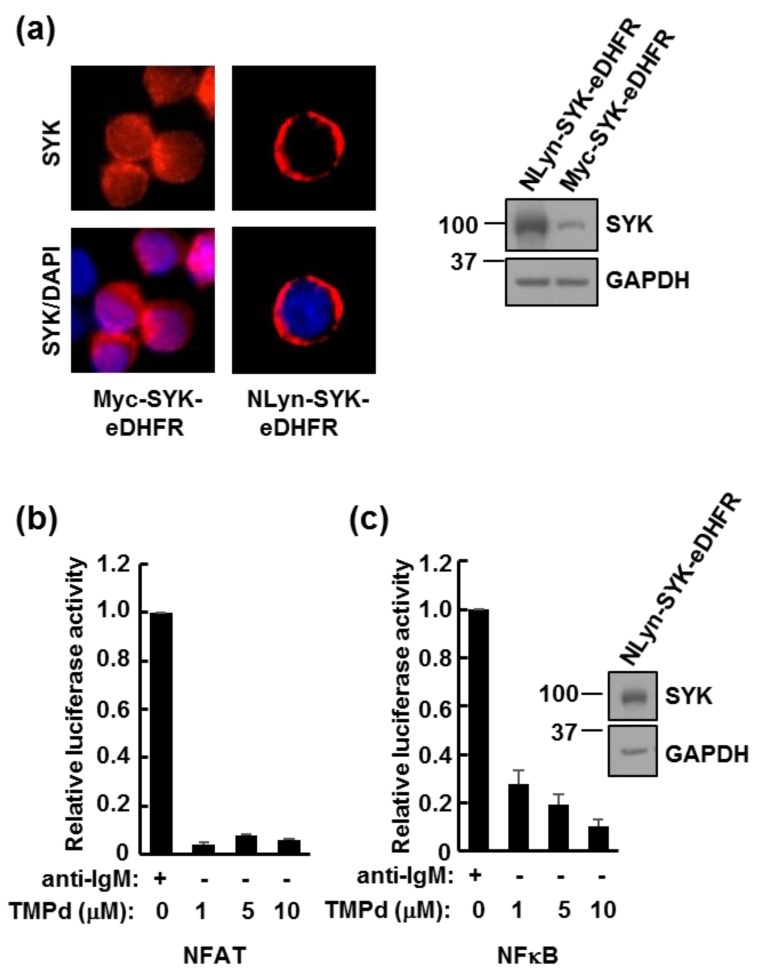
Dimerization of membrane-associated SYK is not sufficient to activate transcription factors. (**a**) SYK-deficient DT40 cells were transiently transfected to express NLyn-SYK-eDHFR or Myc-SYK-eDHFR. Cells were fixed and stained with antibodies against SYK. Nuclei were stained with DAPI. The expression of each protein was verified by Western blotting; (**b**,**c**) SYK-deficient DT40 cells were transiently transfected with plasmids coding for NLyn-SYK-eDHFR and luciferase reporter plasmids for detecting the activation of either Nuclear Factor of Activated T cells (NFAT) (**b**) or NFκB (**c**). Cells were treated with anti-IgM antibodies to crosslink the BCR or with increasing concentrations of trimethoprim (TMP) dimer (TMPd). Data represent the means ± standard error of the mean (SEM) of triplicate experiments repeated three times. The expression of NLyn-SYK-eDHFR was verified by Western blotting with antibodies against SYK.

Previous research has demonstrated that dimerization of SYK by fusion with a dimerization domain derived from TEL results in constitutive kinase activity due to autophosphorylation [37,38]. Therefore, as controls, we asked if the dimerization of Myc-SYK-eDHFR alone was sufficient to trigger signaling through NFAT or NFκB in the absence of BCR crosslinking. At the highest concentration of TMP dimer tested (10 μM), neither transcription factor was activated (Figure 4a,b). We then asked if SYK-eDHFR could transmit signals following dimerization if it was first targeted to the plasma membrane. We expressed in cells a form of SYK-eDHFR fused to a membrane-targeting sequence consisting of the first five amino acids derived from the *N*-terminus of LYN (NLyn). As shown by immunofluorescence of transiently transfected DT40 cells, NLyn-SYK-eDHFR was membrane-bound, while Myc-SYK-eDHFR was expressed throughout the cell (Figure 5a). However, while NLyn-SYK-eDHFR was capable of supporting signaling through the BCR, dimerization alone was still insufficient to propagate a measurable signal (Figure 5b,c). We cannot rule out an activation of SYK-eDHFR fusion proteins through dimerization as is seen with TEL-SYK fusion proteins. However, the TMP dimer does not activate the SYK-fusion protein independent of its interaction with the BCR in a manner that leads to the activation of either NFAT or NFκB.

**Figure 6:**
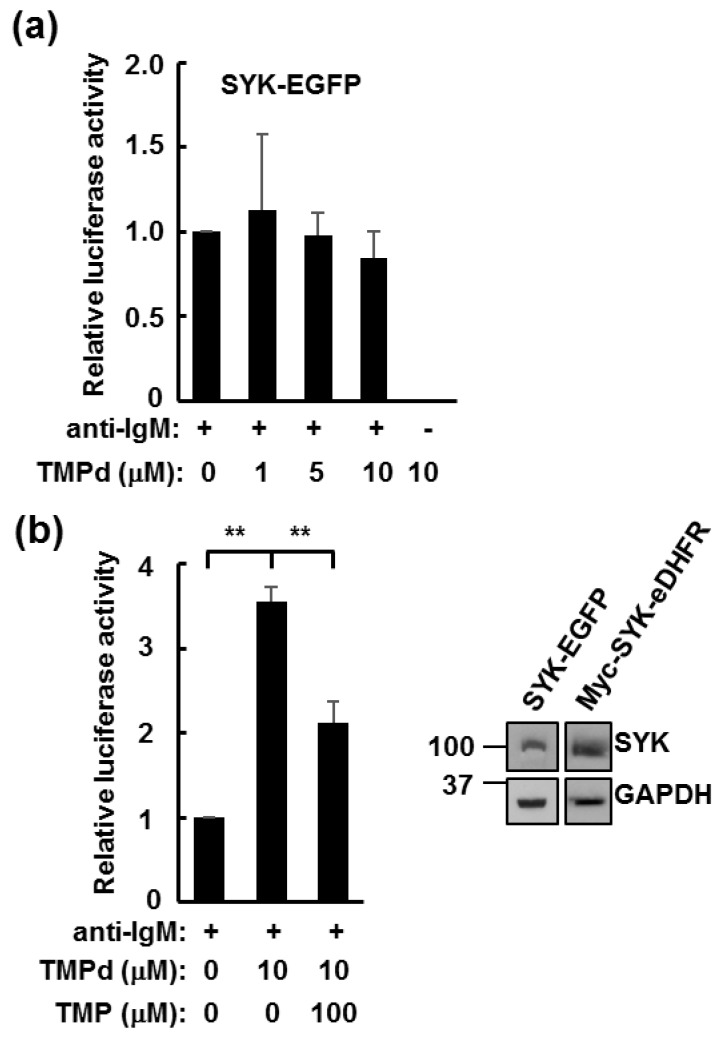
Enhanced Nuclear Factor of Activated T cells (NFAT) activation by the trimethoprim (TMP) dimer requires SYK dimerization. (**a**) SYK-deficient DT40 cells transiently transfected with an NFAT-driven luciferase reporter plasmid and with a plasmid for the expression of SYK-EGFP were activated by receptor crosslinking with anti-IgM where indicated in the presence of increasing concentrations of TMP dimer (TMPd). Values represent means ± standard error of the mean (SEM) of triplicate assays repeated three times; (**b**) SYK-deficient DT40 cells transiently transfected with an NFAT-driven luciferase reporter plasmid and with a plasmid for the expression of Myc-SYK-eDHFR were activated by receptor crosslinking with anti-IgM in the presence of 10 μM TMP dimer (TMPd). Where indicated, an excess of TMP (100 μM) was added 2 h following activation. Values represent means ± SEM of triplicate assays repeated three times. ** *p* < 0.01. The expression of SYK-EGFP and Myc-SYK-eDHFR were verified by Western blotting.

We also performed the same assays with cells expressing SYK lacking the eDHFR affinity tag but containing a different C-terminal tag: EGFP. While cells expressing SYK-EGFP could signal through the BCR to the activation of NFAT, the TMP dimer had no effect on signaling (Figure 6a). Thus, the eDHFR TMP-binding domain was required for dimer-enhanced signaling. As an additional control, we added a ten-fold excess of free TMP two hours after the addition of anti-IgM and TMP dimer to DT40 cells expressing Myc-SYK-eDHFR. Free TMP significantly reduced the dimer-induced increase in NFAT-luciferase activity (Figure 6b). These results indicate that the observed increase in NFAT activity was due to binding of the TMP dimer to eDHFR-tagged SYK.

### 3.4. Dimerization of SYK Promotes Sustained Ca^2+^ Mobilization

**Figure 7:**
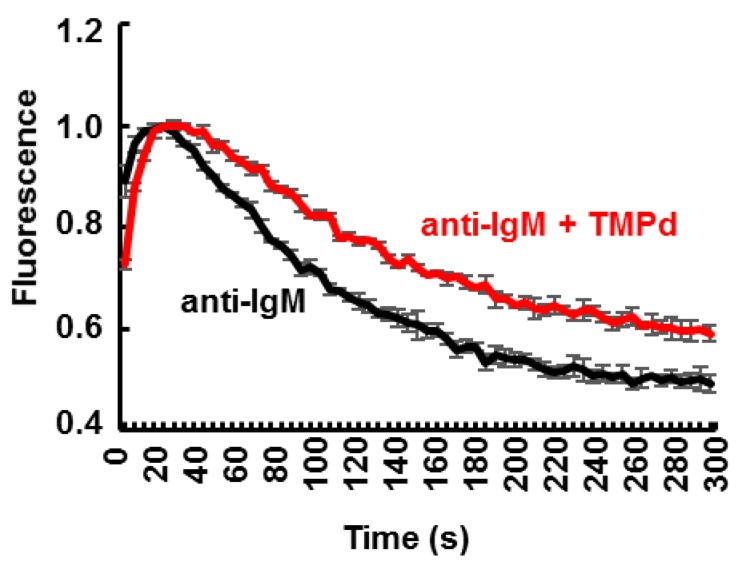
The trimethoprim (TMP) dimer enhances the duration of calcium mobilization. SYK-deficient DT40 cells stably expressing Myc-SYK-eDHFR and labeled with Fluo-4 were activated by anti-IgM in the absence or presence of the TMP dimer (TMPd). Fluorescence readings were obtained every 5 s. Data represent means ± standard error of the mean (SEM) of triplicate experiments repeated three times.

While NFAT and NFκB are both activated by Ca^2+^ signaling downstream of BCR crosslinking, they are differentially regulated by its duration and amplitude. Large, brief pulses of Ca^2+^ activate NFκB, while low, sustained Ca^2+^ mobilization is needed to activate NFAT [39]. To determine the effect of SYK dimerization on Ca^2+^ mobilization, we measured intracellular Ca^2+^ levels in SYK-deficient DT40 B cells stably expressing Myc-SYK-eDHFR. Cells were loaded with the Ca^2+^-binding dye Fluo-4 and activated with or without the TMP dimer. Addition of the TMP dimer resulted in a more sustained mobilization of intracellular Ca^2+^ (Figure 7), an effect that is consistent with the enhanced NFAT activity.

### 3.5. Retention of the BCR at the Plasma Membrane Enhances Signaling through NFAT

**Figure 8:**
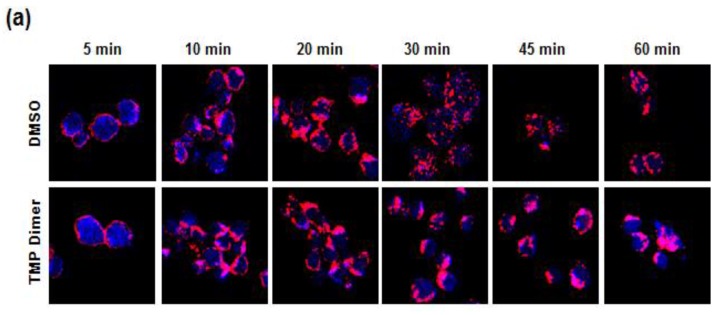

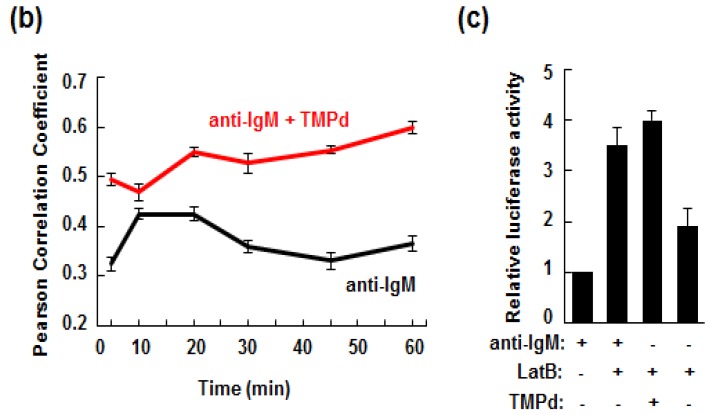
The trimethoprim (TMP) dimer increases the retention of B cell antigen receptor (BCR) complexes. (**a**) DT40 cells stably expressing Myc-SYK-eDHFR were treated with Texas Red-labled anti-IgM (red) with or without TMP dimer for the indicated time points, then fixed, permeabilized and stained with antibodies against SYK (blue); (**b**) Images were analyzed by ImageJ for co-localization of anti-IgM and SYK; (**c**) SYK-dependent DT40 cells transiently transfected with an Nuclear Factor of Activated T cells (NFAT)-driven luciferase reporter plasmid and with a plasmid for the expression of Myc-SYK-eDHFR were activated by receptor crosslinking in the absence or presence of anti-IgM, LatB and/or TMP dimer. Values represent means ± standard error of the mean (SEM) of triplicate assays repeated three times. DMSO, dimethylsulfoxide.

We hypothesized that the prolonged calcium mobilization resulted from a SYK- and dimer-promoted retention of the BCR complex at the plasma membrane. To examine this, we treated DT40 cells stably expressing Myc-SYK-eDHFR with Texas Red-labeled goat anti-chicken IgM for various amounts of time, with or without TMP dimer. We then stained the cells for SYK and analyzed the images for colocalization of the kinase with the BCR at the plasma membrane. Examples of images are shown in Figure 8a. As determined by increased Pearson correlation coefficients, cells treated with the TMP dimer exhibited a larger and more persistent degree of colocalization of SYK with the membrane-bound BCR complex (Figure 8b). 

To further support a role for BCR retention at the membrane in signaling, we inhibited its internalization by promoting actin depolymerization using Latrunculin B (LatB) [25]. The treatment of DT40 cells expressing Myc-SYK-eDHFR with LatB resulted in a substantial increase in anti-IgM-induced NFAT activity (Figure 8c). Consistent with a previous report [22], we found that treatment with the actin depolymerizing agent alone enhanced signaling from the unligated BCR (Figure 8c). This effect was enhanced when the BCR also was crosslinked with anti-IgM. Interestingly, the addition of the TMP dimer also enhanced signaling induced by LatB in the absence of anti-IgM. These data are consistent with a correlation between an enhanced retention of the SYK-BCR complex at the plasma membrane and increased signaling through NFAT.

## 4. Discussion

Several systems have been described for chemically inducing the dimerization of proteins in cells to explore signaling pathways [40]. The first of these utilized a synthetic dimer of FK506 (KF1012) to crosslink a chimeric protein consisting of a myristoylated TCR ζ chain fused to FKBP12 to study TCR signaling [41]. We extended this approach to the development of a methodology for examining the role of SYK and the induced formation of SYK-SYK complexes in BCR-mediated signaling through the generation of a SYK-eDHFR fusion protein and the preparation of a synthetic dimer of TMP. Previous work had shown that eDHFR can be dimerized by a synthetic dimer of methotrexate [42]. For our studies, we generated a dimer of TMP rather than methotrexate since TMP is a specific ligand for the bacterial form of DHFR that is bound with greater than 10,000-fold higher affinity by eDHFR as compared to mammalian DHFR [43]. Fusion proteins containing the eDHFR-tag retain the ability to bind TMP and can be clustered by addition of the TMP dimer as shown by an increase in the size of SGs, which are particles that bind SYK [36] and have liquid-like properties that allow them to fuse to form larger droplets [44].

We find that the induced dimerization of receptor-associated SYK (expressed as Myc-SYK-eDHFR) results in an increased and prolonged existence of kinase-BCR complexes at the plasma membrane. It is clear from studies on antigens and anti-receptor antibodies of different solubilities and valencies that extensive clustering of the BCR complex, or of BCR microclusters, promotes robust signaling [7,8,9,10,11,13,14,19,20]. A critical component of the signal initiation process is the creation of binding sites on the receptor for SYK. BCR engagement also results in receptor internalization but it is the liganded BCR complexes that remain at the plasma membrane that represent the units that are actively signaling [45]. Initial phosphorylation of the first tyrosine of the BCR ITAMs is thought to be catalyzed by LYN [2]. However, continued receptor phosphorylation catalyzed by SYK is needed to maintain active BCR complexes at the membrane. Since BCR internalization requires an unphosphorylated tyrosine residue within the ITAM of CD79B [46], it is likely that continued ITAM phosphorylation by receptor-associated SYK is important for receptor retention at the plasma membrane. Thus, it is likely that the effect of the TMP dimer on signaling in cells expressing Myc-SYK-eDHFR is a consequence of enhanced interactions of neighboring kinase-receptor complexes, leading to their phosphorylation and retention at the plasma membrane.

A role for receptor retention at the membrane for enhancing BCR signaling is consistent with the observation that inhibitors of actin polymerization, which inhibit receptor internalization, also enhance BCR signaling [17,22]. It is interesting that actin depolymerization also can induce signaling from the BCR even in the absence of receptor crosslinking [22] through the opening of BCR receptor complexes that are normally restrained by actin [17]. This actin depolymerization-induced BCR signaling also is dependent on SYK [22], which is required to mediate opening of the BCR oligomers via an inside-out mechanism [17]. Our data indicate that LatB-induced signaling is greatly enhanced by increasing SYK-SYK interactions through kinase dimerization, consistent with a requirement for SYK in mediating BCR signaling induced by actin depolymerization. 

It is interesting that the induced dimerization of receptor-associated SYK affects the quality of the signal sent from the clustered BCR complex. We observe a pronounced effect of kinase dimerization on the receptor-mediated activation of NFAT but no significant effect on the activation of NFκB. SYK, activated at the site of clustered BCR complexes, catalyzes the phosphorylation of the scaffolding protein BLNK/SLP-65. SYK also has been reported to physically associate with BLNK such that a kinase-independent scaffolding function also contributes to enhanced Ca^2+^ mobilization [47]. This SYK-dependent phosphorylation of BLNK promotes the assembly of signalosome complexes containing BTK and PLCγ. PLCγ, activated by BTK, is essential for the generation of the second messengers, diacylglycerol and inositol trisphosphate, needed for calcium mobilization and activation of both the NFAT and NFκB pathways [48]. The activation of NFκB occurs in response to high but transient, increases in intracellular calcium, while NFAT is activated in response to low but sustained levels of calcium [39]. A selective activation of NFAT is consistent with our observed changes in the duration but not the amplitude of calcium responses that we see in cells that are treated with anti-IgM in the presence of the TMP dimer. This prolonged maintenance of the calcium plateau in dimer-treated cells is likely a consequence of the prolonged residency of kinase-BCR complexes at the membrane. Thus, molecules and processes that regulate receptor clustering and internalization from the interior of the cell have the capacity to modulate the quality of signals sent from the antigen receptor. This is interesting as the physiological outcome of receptor occupancy, cellular activation versus anergy, also is dependent on the quality of the signal, with prolonged receptor occupancy and selective activation of NFAT being associated with anergic responses [49,50].
